# The weekend effect in liver transplantation

**DOI:** 10.1371/journal.pone.0198035

**Published:** 2018-05-24

**Authors:** Felix Becker, Thomas Vogel, Thekla Voß, Anne-Sophie Mehdorn, Katharina Schütte-Nütgen, Stefan Reuter, Annika Mohr, Iyad Kabar, Eike Bormann, Thorsten Vowinkel, Daniel Palmes, Norbert Senninger, Ralf Bahde, Linus Kebschull

**Affiliations:** 1 Department of General, Visceral and Transplant Surgery, University Hospital Münster, Münster, Germany; 2 Department of Internal Medicine D, Division of General Internal Medicine, Nephrology and Rheumatology, University Hospital Münster, Münster, Germany; 3 Department of Internal Medicine B, Gastroenterology and Hepatology, University Hospital Münster, Münster, Germany; 4 Institute of Biostatistics and Clinical Research, University Hospital Münster, Münster, Germany; Medizinische Universitat Graz, AUSTRIA

## Abstract

**Background:**

The weekend effect describes a phenomenon whereby patients admitted to hospitals on weekends are at higher risk of complications compared to those admitted during weekdays. However, if a weekend effect exists in orthotopic liver transplantation (oLT).

**Methods:**

We analyzed oLT between 2006 and 2016 and stratified patients into weekday (Monday to Friday) and weekend (Saturday, Sunday) groups. Primary outcome measures were one-year patient and graft survival.

**Results:**

364 deceased donor livers were transplanted into 329 patients with 246 weekday (74.77%) and 83 weekend (25.23%) patients. Potential confounders (e.g. age, ischemia time, MELD score) were comparable. One-year patient and graft survival were similar. Frequencies of rejections, primary-non function or re-transplantation were not different. The day of transplantation was not associated with one-year patient and graft survival in multivariate analysis.

**Conclusions:**

We provide the first data for the Eurotransplant region on oLT stratified for weekend and weekday procedures and our findings suggest there was no weekend effect on oLT. While we hypothesize that the absent weekend effect is due to standardized transplant procedures and specialized multidisciplinary transplant teams, our results are encouraging showing oLT is a safe and successful procedure, independent from the day of the week.

## Introduction

The weekend effect describes a phenomenon whereby patients admitted to hospitals on weekends are at higher risk of complications, worse outcome and death compared to those admitted during weekdays [1]. Previous studies suggested a reduction in medical staffing and resources as well as a possible restriction in diagnostic and therapeutic tools on weekends to be responsible for the observed differences in mortality and morbidity [2, 3]. While studies have shown a weekend effect for different medical conditions (including acute kidney injury [4], pneumonia [5] or dysrhythmia [6]) major commonalities among conditions affected by a weekend effect are the need for time sensitive interventions and care at an intensive care unit. In line with this, there are numerous reports about a weekend effect for stroke [7–9] myocardial infarction [10, 11] or pulmonary embolism [12, 13]. Additionally, a growing body evidence points towards a weekend effect for conditions requiring emergency surgery e.g. ruptured aortic aneurysms [14] as well as emergency procedures in general surgery such as laparotomy, partial colectomy or small bowel resection [15].

Solid organ transplantation and especially orthotopic liver transplantation (oLT) appears to be prone to a weekend effect since it combines both time sensitive and urgent procedures, as well as postoperative care at an intensive care unit. However, it remains unknown if the weekend effect applies to oLT. On the one hand, deceased donor oLT cannot be scheduled since its time point depends upon organ availability and timing of organ procurement. On the other hand, these procedures are regularly performed by small, highly specialized teams, postoperative care is standardized and in the hand of specialized multidisciplinary units, where fluctuations in staffing levels are minimal.

Recent data from the United Network of Organ Sharing (UNOS) database suggest that there is no weekend effect in oLT when comparing one-year graft and patient survival [16]. However, based on the large database design of this study, important functional outcome parameters (such as episodes of acute rejections, rates of primary non-function, frequencies of re-transplantations) as well as surrogate markers for the quality of surgery (complications and reoperations) are missing. Therefore, we conducted a retrospective single center study in a German transplant center within the Eurotransplant region, which aimed to investigate whether outcomes were similar for weekday versus weekend oLT considering the above-mentioned outcome parameters.

## Methods

### Patients

The study design was a retrospective single center study of transplant outcomes at the transplant center of the University Hospital Münster. We analyzed data from 329 oLT patients, who underwent deceased donor oLT or combined deceased donor multi-organ transplant (including a liver graft) between January 2006 and June 2016 at our center. Patients were grouped based on the time of oLT, with weekend oLT being defined as Saturday/Sunday procedures whereas procedures performed from Monday to Friday counted as weekday oLT. The following donor parameters were extracted and analyzed from the Eurotransplant Network Information System (ENIS): age, gender, body mass index (BMI) and donor center (national/international). For recipients we examined patient demographics (age, gender, BMI), model for end-stage liver disease (MELD) score, indication for oLT, high urgency (HU) status, time on waiting list, cold and warm ischemia time, numbers of prior transplants and frequencies of combined transplantations. Retransplantations within one year were not counted as additional oLT cases. However, they were counted as a complication for the respective group of the initial oLT (weekend or weekday).

Approval to conduct this retrospective study was obtained from the local ethics committee and institutional review board (Ärztekammer Westfalen-Lippe and Medizinischen Fakultät der Westfälischen Wilhelms-Universität, No. 2014-381-f-N). All participants had given written informed consent to record their clinical data. Recipient and donor data were all collected from patients’ charts, ENIS or in-house transplant data files and de-identified prior to analysis. This study was carried out in accordance with the Declarations of Istanbul and Helsinki.

### Outcome measures

Follow-up time was 12 months and primary outcome measures were one-year patient, death-censored graft and overall graft survival. Secondary outcome parameters were 30-day and 90-day patient and graft survival, rates of biopsy proven acute rejections (AR), primary non-function (PNF, defined as graft failure resulting in death or re-transplantation within 30 days of the initial transplant excluding any identifiable cause of graft failure such as rejection or vascular thrombosis), rates of re-transplantations, peak serum values of aspartate transaminase (AST) and alanine transaminase (ALT), length of stay at the intensive care unit (ICU), length of stay in the hospital, death within the initial stay as well as the number and length of re-admissions after initial hospital discharge. We also examined surgical complications which required reoperation (excluding re-transplantations). They were further categorized in 1) haemorrhage (defined as any hematoma or bleeding related to the transplant procedure), 2) vascular complications (hepatic artery stenosis or thrombosis, portal vein thrombosis, hepatic venous obstruction), 3) biliary tract complications (stricture, leak, fistula and T-tube dislocation), 4) wound complications (impaired wound healing or dehiscence), 5) gastrointestinal complications (ulcer, perforation, bleeding and anastomic leakage) and 6) other complications related to the transplant procedure which required surgical intervention.

### Statistical analysis

Statistical analysis was performed using IBM SPSS® Statistics 22 for Windows (IBM Corporation, Somers, NY, USA). Normally distributed continuous variables are shown as mean ± standard deviation (SD) and not normally distributed continuous variables are presented as median and quartiles (interquartile range, IQR, Q_0.25_—Q_0.75_). Groups were compared using Student’s *t*-test for normally distributed data, Mann-Whitney U test for not normally distributed data and Fisher's exact test for categorical variables. Cox proportional hazards regression models were constructed to assess associations between weekend or weekday oLT and the primary outcomes one-year patient survival, death-censored graft survival and overall graft survival, while simultaneously adjusting for potential confounders. Univariate analysis included weekend or weekday status, recipient age, gender and BMI, cold and warm ischemia time, MELD, time on waiting list, HU status, prior and combined transplantations, donor age, gender, BMI and donor center as well as PNF, AR, peak AST and ALT, stay at ICU, initial hospital stay, number and length of readmissions, reoperations and re-transplantations. Using a stepwise variable selection procedure for covariates with a *p*-value less than 0.05 the multivariable logistic regression analysis for one-year patient survival, death-censored graft survival and overall graft survival were adjusted for PNF, stay at ICU, number and length of readmissions and reoperations, respectively. Results are shown as hazard ratios (HR) with 95% confidence interval (CI) and *p*-value of likelihood ratio test. Patient survival, death-censored graft survival and overall graft survival were analyzed using the Kaplan-Meier method, and the two groups were compared by log-rank test, *p-*values less than 0.05 were considered statistically significant.

## Results

### Study population characteristics

During the study period from January 2006 to June 2016, 364 deceased donor livers were transplanted into 329 patients at our center. 246 patients (74.7%) underwent oLT on a weekday and 83 (25.2%) during a weekend. When analyzed for recipient baseline characteristics no differences were observed for age, gender or BMI. (Table 1) Hepatocellular carcinoma was the leading indication for oLT in both the weekday (22%) and weekend (24.1%) group. Combined, 90.58% of all oLTs were conducted in the MELD era with a mean MELD score at oLT of 22.2±12.2 for weekday and 22.1±12 for weekend patients. There was no difference in the outcome measures when MELD and Non-MELD groups were separately stratified for weekday and weekend procedures and analyzed individually. Average time on the waiting list was 120.5 days (IQR 18–313.8) for weekday and 198.5 days (IQR 47–456) for weekend patients. The two groups were approximately similar with respect to the number of HU patients, combined transplant procedures as well as patients with prior transplants. (Table 1) When analyzed for donor baseline characteristics no differences were observed for age, gender or BMI. (Table 2) For weekday oLT, 87.4% of the organs were procured at national donor centers, whereas for weekend oLT 95.2% of all grafts came from national centers. (Table 2) This difference was not significant (*p* = 0.06) and had no influence on cold ischemia time (weekday: 10.1±2.6h, weekend: 9.8±2.4h). In addition, warm ischemia times were similar between both groups. (Table 1)

**Table 1:** Recipient characteristics.

	Weekday oLT (n = 246)	Weekend oLT (n = 83)	*p*-value
**Age** *(years*, *mean ± SD)*	53.1 ± 10.8	51.4 ± 11.7	0.208^a^
**Gender** *(% males)*	67.9	56.6	0.083^b^
**BMI** *(kg/m*^*2*^, *median (Q*_*0*.*25*_, *Q*_*0*.*75*_*))*	25.6 (22.8–29.4)	24.0 (22.0–28.1)	0.026^c^
**Indication for transplant (%)**			0.312^b^
ALF	13.4	8.4	
HCC	22.0	24.1	
Viral Hepatitis	14.6	9.6	
PSC, PBC, SSC	8.1	13.3	
Alcoholic Cirrhosis	15.0	18.1	
Cirrhosis other	7.3	2.4	
PLD	4.5	7.2	
Other	15.0	16.9	
**Cold ischemia time** *(h*, *mean ± SD)*	10.1 ± 2.6	9.8 ± 2.4	0.371^a^
**Warm ischemia time** *(min*, *mean ± SD)*	41.3 ± 9.4	41.8 ± 8.9	0.722^a^
**MELD** *(mean ± SD)*	22.2 ± 12.2	22.1 ± 12.0	0.962^a^
**Time on waiting list** *(d*, *median (Q*_*0*.*25*_*—Q*_*0*.*75*_*))*	120.5 (18.0–313.8)	198.5 (47.0–456.0)	0.099^c^
**HU Status** *(% HU)*	4.9	3.6	0.769^b^
**≥ 1 prior transplant** *(n*, *%)*	27 (11.0)	9 (10.8)	1.000^b^
**Combined Tx** *(n*, *%)*	16 (6.5)	7 (8.4)	0.619^b^
Combined kidney *(n*, *% of combined Tx)*	10 (62.5)	6 (85.7)	
Combined pancreas *(n*, *% of combined Tx)*	2 (12.5)	0 (0.0)	
Combined heart *(n*, *% of combined Tx)*	0 (0.0)	1 (14.3)	
Combined small bowel *(n*, *% of combined Tx)*	4 (25.0)	0 (0.0)	
**AB0 blood group** *(%)*			0.078^b^
A	46.7	44.6	
AB	4.1	10.8	
B	15.0	8.4	
0	34.1	36.1	

Baseline comparison of recipient characteristics in patients with orthotopic liver transplantation (oLT) stratified by weekday and weekend transplantation. BMI = body mass index, ALF = acute liver failure, HCC = hepatocellular carcinoma, PSC = primary sclerosing cholangitis, PBC = primary biliary cholangitis, SSC = secondary sclerosing cholangitis, PLD = polycystic liver disease, MELD = model for end-stage liver disease, HU = high urgency, Tx = transplantation.

^a^) Student’s *t*-test

^b^) Fisher's exact test

^c^) Mann-Whitney U test, a *p*-value less than 0.05 was considered statistically significant.

**Table 2:** Donor characteristics.

	Weekday oLT (n = 246)	Weekend oLT (n = 83)	*p*-value
**Age** *(years*, *mean ± SD)*	50.8 ± 15.4	52.9 ± 13.9	0.272^a^
**Gender** *(% males)*	59.3	57.8	0.897^b^
**BMI** *(kg/m*^*2*^, *median (Q*_*0*.*25*_*—Q*_*0*.*75*_*))*	25.0 (23–1, 27.8)	25.7 (24.2–27.8)	0.222^c^
**Donor center** *(% national)*	87.4	95.2	0.062^b^
**DRI** *(mean ± SD)*	1.7 ± 0.3	1.8 ± 0.3	0.165^a^

Baseline donor characteristics stratified by weekday and weekend orthotopic liver transplantation. BMI = body mass index, DRI = donor risk index.

^a^) Student’s *t*-test

^b^) Fisher's exact test and

^c^) Mann-Whitney U test, a *p*-value less than 0.05 was considered statistically significant.

### One-year patient and graft survival

**Fig 1:**
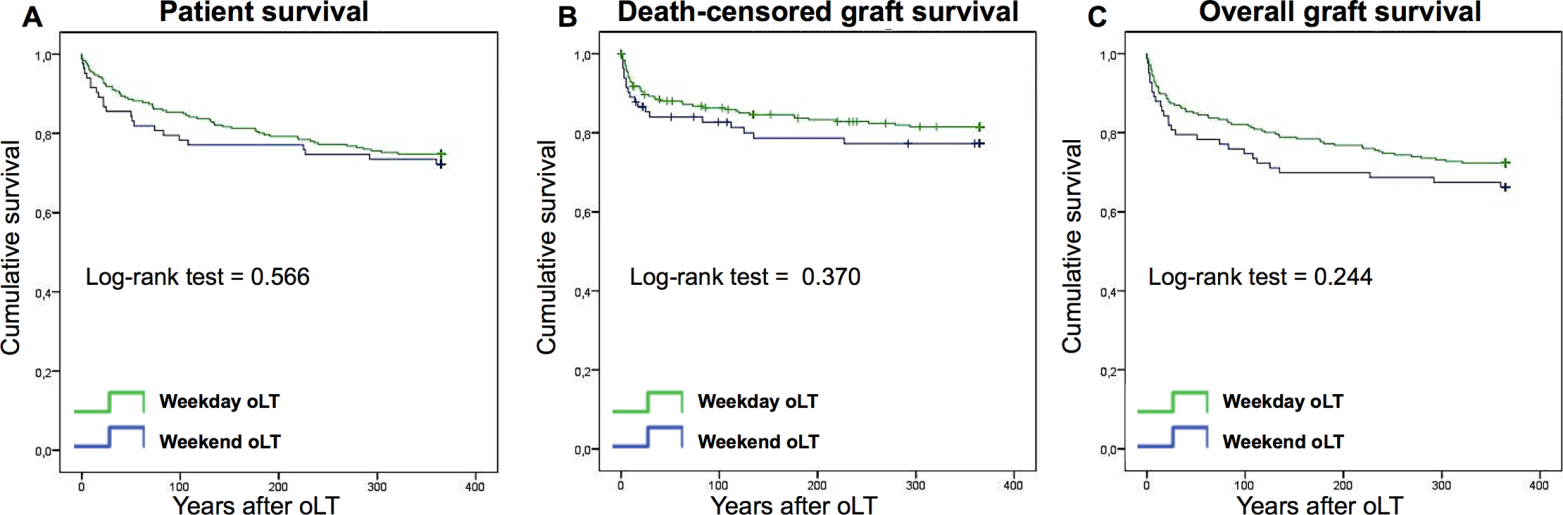
Kaplan-Meier curves for one-year patient and graft survival. Longitudinal survivals of patient survival (**A**), death-censored graft survival (**B**) and overall graft survival (**C**) separated for weekday and weekend orthotopic liver transplantation (oLT). Survival rates of weekday (green lines) and weekend (blue lines) oLT recipients were estimated by Kaplan-Meier methodology and compared by log-rank test.

The primary outcome measures were one-year patient survival, death-censored graft survival and overall graft survival. Kaplan-Meier analysis was used to generate survival curves for one-year patient (Fig 1A), death-censored graft survival (Fig 1B) and overall graft survival (Fig 1C) for weekday and weekend oLT as shown in Fig 1. When groups were compared by log-rank test, no differences were found. Similarly, no statistical significant differences in patient survival were seen at 30 (weekday 91.9%, weekend 85.5%, *p* = 0.09), 90 (weekday 85%, weekend 79.5%, *p* = 0.23) and 365 (weekday 74.8%, weekend 72.3%, *p* = 0.57) days as well as in graft survival at 30 (weekday 87%, weekend 79.5%, *p* = 0.1), 90 (weekday 81.7%, weekend 75.9%, *p* = 0.23) and 365 (weekday 72.4%, weekend 66.3%, *p* = 0.24) days, respectively (Table 3). Occurrence of death within the first 30 days following oLT (weekday: 8.1%, weekend 14.5%, *p* = 0.13) as well as death within the initial hospital admission (weekday: 17.1%, weekend 20.5%, *p* = 0.51) were also similar between the two groups. (Table 3) Unadjusted Cox proportional hazard modeling showed that weekend oLT patients had a 0.870 (0.539–1.403 95% CI, Table 4) hazard of death at 365 days, a 0.779 (0.450–1.348 95% CI, Table 5) hazard of death-censored graft loss and a 0.771 (0.496–1.197 95% CI, Table 6) hazard of overall graft loss. Similar results were obtained in a Cox regression analysis adjusted for potential confounders. The day of transplantation (weekday vs weekend) was not associated with one-year patient survival, death-censored and overall graft survival in the multivariate analysis.

**Table 3:** Results.

	Weekday oLT (n = 246)	Weekend oLT (n = 83)	*p*-value
**Patient survival** *(%)*			
30 d	91.9	85.5	0.090^c^
90 d	85.0	79.5	0.227^c^
1 y	74.8	72.3	0.566
**Graft survival** *(%)*			
30 d	87.0	79.5	0.099^c^
90 d	81.7	75.9	0.230^c^
1 y	72.4	66.3	0.244^c^
**Retransplantation within 1 y** *(%)*	8.9	10.8	0.664^a^
**PNF** *(%)*	6.9	12.1	0.165^a^
**Biopsy proven rejection** *(%)*	14.6	19.3	0.384^a^
**Peak AST** *(U/l*, *median (Q*_*0*.*25*_*—Q*_*0*.*75*_*))*	3442.0 (1666.0–8614.0)	4380.0 (1253.0–9122.0)	0.797^b^
**Peak ALT** *(U/l*, *median (Q*_*0*.*25*_*—Q*_*0*.*75*_*))*	2072.0 (929.0–4882.0)	2175.0 (805.0–4973.0)	0.940^b^
**Stay at ICU** *(d*, *median (Q*_*0*.*25*_*—Q*_*0*.*75*_*))*	6.0 (3.0–18.5)	5.0 (2.0–11.0)	0.048^b^
**Initial hospital stay** *(d*, *median (Q*_*0*.*25*_*—Q*_*0*.*75*_*))*	38.5 (21.8–70.3)	30.0 (18.0–59.0)	0.055^b^
**Death within 30 d** *(%)*	8.1	14.5	0.131^a^
**Death within initial stay** *(%)*	17.1	20.5	0.509^a^
**Number of readmissions** *(median (min*, *max))*	1.0 (0, 10)	1.0 (0, 8)	0.912^b^
**Length of readmissions** *(d*, *median (min*, *max))*	23.0 (1, 219)	27.0 (1, 119)	0.961^b^
**Reoperation** *(%)*	56.9	59.0	0.798^a^
**Number of reoperations** *(median (min*, *max))*	1.0 (1, 53)	1.0 (1, 14)	0.976^b^
**Indications for reoperation** *(%)*			
Haemorrhage	25.0	29.7	0.903^b^
Vascular complications	8.8	11.6	0.455^b^
Biliary tract complications	17.6	22.4	0.737^b^
Wound complications	16.4	8.0	0.327^b^
Gastrointestinal complications	13.8	5.1	0.923^b^
Other	18.4	23.2	0.804^b^

Primary and secondary outcomes for orthotopic liver transplantation (oLT) stratified by weekday and weekend status. PNF = primary non-function, AST = aspartate aminotransferase, ALT = alanine aminotransferase, ICU = intensive care unit.

^a^) Fisher's exact test

^b^) Mann-Whitney U test and

^c^) Log-rank test, a *p*-value less than 0.05 was considered statistically significant.

**Table 4:** Cox regression model for one-year patient survival.

	Univariate HR (95% CI) *p*-value	Multivariate HR (95% CI) *p*-value
**Weekend transplant status** *(weekday vs weekend)*	0.870 (0.539–1.403) 0.567	
**Recipient age** *(years)*	1.006 (0.986–1.026) 0.556	
**Recipient gender** *(male vs female)*	1.017 (0.652–1.587) 0.940	
**Recipient BMI** *(kg/m*^*2*^*)*	0.985 (0.945–1.026) 0.468	
**Cold ischemia time** *(hours)*	0.999 (0.918–1.086) 0.977	
**Warm ischemia time** *(minutes)*	1.006 (0.983–1.030) 0.620	
**MELD**	1.029 (1.009–1.049) 0.004	
**Time on waiting list** *(days)*	1.000 (0.999–1.000) 0.200	
**HU Status** *(yes vs no)*	0.486 (0.212–1.114) 0.088	
**≥ 1 prior transplant** *(yes vs no)*	0.787 (0.418–1.483) 0.459	
**Combined Tx** *(yes vs no)*	0.709 (0.342–1.469) 0.355	
**Donor age** *(years)*	1.005 (0.991–1.020) 0.476	
**Donor gender** *(male vs female)*	1.213 (0.791–1.861) 0.375	
**Donor BMI** *(kg/m*^*2*^*)*	0.985 (0.935–1.038) 0.578	
**Donor Center** *(national vs international)*	1.049 (0.526–2.094) 0.892	
**Re-oLT within 1y** *(yes vs no)*	0.248 (0.150–0.411) <0.001	
**PNF** *(yes vs no)*	0.113 (0.068–0.187) <0.001	
**Biopsy proven rejection** *(yes vs no)*	0.897 (0.513–1.566) 0.701	
**Peak AST** *(U/l)*	1.000 (1.000–1.000) 0.361	
**Peak ALT** *(U/l)*	1.000 (1.000–1.000) 0.905	
**Stay at ICU** *(days)*	1.014 (1.010–1.018) <0.001	
**Initial hospital stay** *(days)*	1.003 (1.000–1.006) 0.098	
**Number of readmissions**	0.409 (0.316–0.529) <0.001	0.417 (0.316–0.550) <0.001
**Length of readmissions** *(days)*	0.979 (0.967–0.990) <0.001	
**Reoperation** *(yes vs no)*	0.205 (0.113–0.370) <0.001	0.199 (0.090–0.441) <0.001
**Number of reoperations**	1.053 (1.032–1.073) <0.001	1.105 (1.024–1.193) 0.010

Cox proportional hazards regression model with univariate and multivariable logistic regression analysis of patient survival. HR = hazard ratios, CI = 95% confidence interval. MELD = model for end-stage liver disease, BMI = body mass index, PNF = primary non-function, HU = high urgency, Tx = transplantation, AST = aspartate aminotransferase, ALT = alanine aminotransferase, ICU = intensive care unit

**Table 5:** Univariate and multivariate analyses of death-censored graft survival.

	Univariate HR (95% CI) *p*-value	Multivariate HR (95% CI*) p*-value
**Weekend transplant status** *(weekday vs weekend)*	0.779 (0.450–1.348) 0.372	
**Recipient age** *(years)*	1.000 (0.978–1.024) 0.970	
**Recipient gender** *(male vs female)*	0.842 (0.490–1.444) 0.531	
**Recipient BMI** *(kg/m*^*2*^*)*	1.008 (0.964–1.054) 0.736	
**Cold ischemia time** *(hours)*	0.983 (0.890–1.087) 0.741	
**Warm ischemia time** *(minutes)*	1.003 (0.976–1.031) 0.813	
**MELD**	1.022 (0.999–1.046) 0.057	
**Time on waiting list** *(days)*	1.000 (0.999–1.000) 0.632	
**HU Status** *(yes vs no)*	0.283 (0.129–0.622) 0.002	
**≥ 1 prior transplant** *(yes vs no)*	1.154 (0.497–2.679) 0.739	
**Combined Tx** *(yes vs no)*	1.121 (0.407–3.088) 0.825	
**Donor** *age (years)*	1.000 (0.984–1.017) 0.983	
**Donor gender** *(male vs female)*	1.259 (0.764–2.077) 0.366	
**Donor BMI (kg/m**^**2**^**)**	0.948 (0.886–1.013) 0.114	
**Donor Center** *(national vs international)*	1.092 (0.497–2.397) 0.827	
**PNF** *(yes vs no)*	0.011 (0.005–0.023) <0.001	0.037 (0.017–0.080) <0.001
**Biopsy proven rejection** *(yes vs no)*	1.517 (0.691–3.331) 0.299	
**Peak AST** *(U/l)*	1.000 (1.000–1.000) 0.005	1.000 (1.000–1.000) 0.045
**Peak ALT***(U/l)*	1.000 (1.000–1.000) 0.072	
**Stay at ICU** *(days)*	1.013 (1.009–1.018) <0.001	1.014 (1.005–1.024) 0.003
**Initial hospital stay** *(days)*	1.004 (1.001–1.007) 0.021	
**Number of readmissions**	0.427 (0.318–0.572) <0.001	0.587 (0.435–0.793) <0.001
**Length of readmissions** *(days)*	0.978 (0.965–0.992) 0.002	
**Reoperation** *(yes vs no)*	0.077 (0.028–0.213) <0.001	0.166 (0.058–0.477) <0.001
**Number of reoperations**	1.057 (1.036–1.079) <0.001	

Cox proportional hazards regression model with univariate and multivariable logistic regression analysis of death-censored graft survival. HR = hazard ratios, CI = 95% confidence interval, MELD = model for end-stage liver disease, BMI = body mass index, PNF = primary non-function, HU = high urgency, Tx = transplantation, AST = aspartate aminotransferase, ALT = alanine aminotransferase, ICU = intensive care unit.

**Table 6:** Univariate and multivariate analyses of overall graft survival.

	Univariate HR (95% CI) *p*-value	Multivariate HR (95% CI) *p*-value
**Weekend transplant status** *(weekday vs weekend)*	0.771 (0.496–1.197) 0.246	
**Recipient age** *(years)*	0.998 (0.980–1.017) 0.839	
**Recipient gender** *(male vs female)*	1.097 (0.724–1.662) 0.662	
**Recipient BMI** *(kg/m*^*2*^*)*	0.984 (0.947–1.023) 0.424	
**Cold ischemia time** *(hours)*	0.988 (0.912–1.071) 0.774	
**Warm ischemia time** *(minutes)*	1.002 (0.980–1.024) 0.880	
**MELD**	1.025 (1.007–1.044) 0.007	
**Time on waiting list** *(days)*	1.000 (0.999–1.000) 0.477	
**HU Status** *(yes vs no)*	0.447 (0.207–0.966) 0.040	
**≥ 1 prior transplant** *(yes vs no)*	0.951 (0.507–1.782) 0.875	
**Combined Tx** *(yes vs no)*	0.844 (0.409–1.741) 0.647	
**Donor age** *(years)*	1.004 (0.990–1.018) 0.579	
**Donor gender** *(male vs female)*	1.358 (0.910–2.028) 0.134	
**Donor BMI** *(kg/m*^*2*^*)*	0.969 (0.921–1.020) 0.229	
**Donor Center** *(national vs international)*	0.887 (0.447–1.762) 0.733	
**PNF** *(yes vs no)*	0.021 (0.011–0.040) <0.001	0.073 (0.036–0.146) <0.001
**Biopsy proven rejection** *(yes vs no)*	0.889 (0.526–1.501) 0.659	
**Peak AST** *(U/l)*	1.000 (1.000–1.000) 0.066	
**Peak ALT** *(U/l)*	1.000 (1.000–1.000) 0.440	
**Stay at ICU** *(days)*	1.013 (1.009–1.017) <0.001	1.007 (1.001–1.013) 0.015
**Initial hospital stay** *(days)*	1.003 (1.000–1.006) 0.038	
**Number of readmissions**	0.481 (0.391–0.592) <0.001	0.480 (0.367–0.628) <0.001
**Length of readmissions** *(days)*	0.983 (0.973–0.993) <0.001	1.008 (1.000–1.016) 0.046
**Reoperation** *(yes vs no)*	0.169 (0.094–0.304) <0.001	0.172 (0.080–0.370) <0.001
**Number of reoperations**	1.053 (1.034–1.072) <0.001	

Cox proportional hazards regression model with univariate and multivariable logistic regression analysis of overall graft survival. HR = hazard ratios, CI = 95% confidence interval. MELD = model for end-stage liver disease, BMI = body mass index, PNF = primary non-function, HU = high urgency, Tx = transplantation, AST = aspartate aminotransferase, ALT = alanine aminotransferase, ICU = intensive care unit.

### Re-transplantation, liver function and complications

In line with the primary outcome measures, no statistical significant difference was found in the secondary outcome measures in the present study. Rates of retransplantation within the first year were similar between weekday (8.9%) and weekend (10.8%, *p* = 0.66) oLT. Frequencies of PNF were comparable between patients who underwent oLT on a weekday (6.9%) or weekend (12.1%, *p* = 0.17) and following weekday oLT peak values of liver enzymes (AST, ALT) were comparable to weekend oLT (*p* = 0.8 and 0.94, respectively). Frequency of patients experiencing ≥ 1 surgical complication which required reoperation within the first year after oLT was comparable between weekday (56.9%) and weekend (59.0%, *p* = 0.8) oLT. In addition, the median number of reoperation was similar between the two groups. The most common surgical complications in both groups were hemorrhagic complications, followed by biliary and wound complications. Comparing the median length of stay at the ICU following oLT, a significant (*p* = 0.05) shorter stay was found for weekend procedures (5 days, IQR 2–11) compared to weekday procedures (6 days, IQR 3–18.5). Although it was not statistical significant this trend was also observed when analyzing the median length of the initial hospital stay after transplantation (weekday: 38.5 days, IQR 21.8–70.3; weekend: 30 days, IQR 18–59, *p* = 0.06) as well as the length of stay following readmission (weekday: 23 days, range 1–219; weekend: 27 days, range 1–119, *p* = 0.96). (**Table 3**)

**Fig 2:**
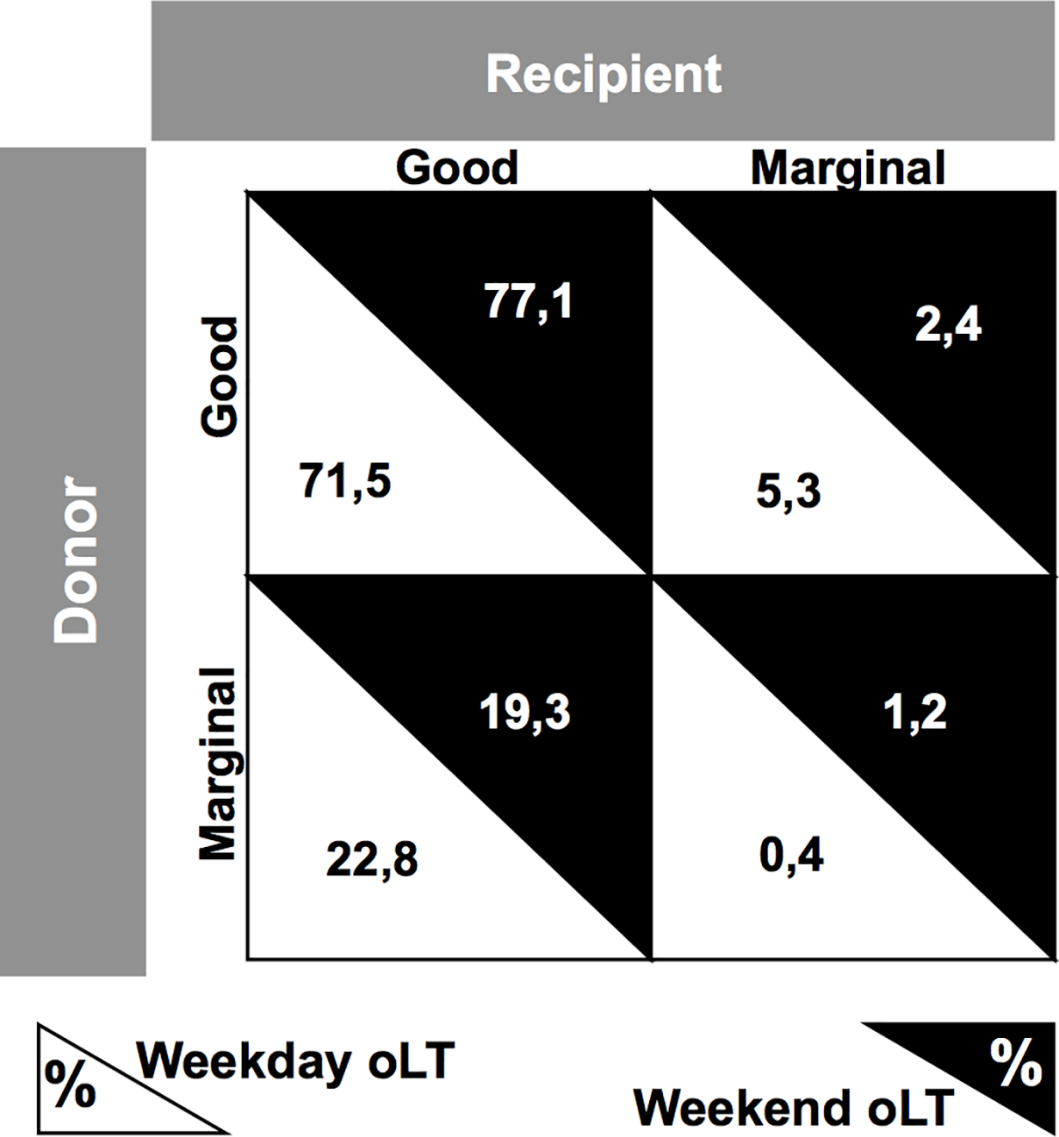
Match frequencies of marginal livers and marginal recipients. Analysis of match frequencies for marginal livers and marginal recipients in weekday and weekend orthotopic liver transplantation (oLT). Marginal livers were defined as donor age > 60 years, BMI > 25 kg/m^2^ and cold ischemia time > 12 hours, while marginal recipients were defined those with a MELD > 35 or HU-status.

Finally, we analyzed a potential allocation bias by comparing match frequencies of marginal livers and marginal recipients. Marginal livers were defined as donor age > 60 years [17], BMI > 25 kg/m^2^ [18] and cold ischemia time > 12 hours [19], while marginal recipients were defined as those with a MELD > 35 or HU-status [20]. In addition, we calculated the donor risk index (DRI, [17]) for every patient. No statistical significant differences (*p* = 0.44) were found when comparing matching frequencies of marginal liver grafts and marginal recipients in weekend (1.2%) and weekday (0.4%) oLT. Furthermore, no statistical significant differences (p = 0.16) were found when comparing the DRI for weekday (1.7 ± 0.3) and weekend (1.8 ± 0.3) donors. (Fig 2)

## Discussion

One of the undisputed dogmas of transplant medicine is the reduction of cold ischemia time by any means. Timing of procedures such as oLT is therefore mainly driven by donor organ availability and time of organ procurement since especially for liver grafts the acceptable window of cold ischemia time is fairly small. Thus, oLT is often performed during off-hours, such as weekends. It is now recognized that the outcome for various time-sensitive or emergency medical conditions shows an association between weekend hospital admissions and increased rates of morbidity and mortality [1]. However, there is only limited data analyzing this so-called weekend effect in transplant medicine.

There are neither specific reports of a weekend effect in thoracic transplantation nor any data on intestinal or pancreas transplantation. Regarding adult deceased donor single kidney transplantation, two large studies (combining 149,617 patients, 136,715 from the US [21] and 12,902 from the UK [22] could recently demonstrate a similar one-year patient and graft survival between weekend and weekday patients. The US study by Anderson et al. also reported comparable secondary outcomes, including delayed graft function or acute rejection [22]. Similar to our study, Baid-Agrawal et al. found a trend towards a shorter hospital stay in the weekend group [21]. Furthermore, our group recently reported a possible weekend effect in adult deceased donor single kidney transplantation [23].

Regarding a possible weekend effect in oLT, there is currently only one study from Orman et al. analyzing 94,768 liver transplants between 1987 and 2010 using the UNOS database [16]. The authors revealed a slight increase in one-year allograft failure in the weekend group, but found no influence of the day of surgery on one-year patient survival. However, there are important distinctions between the work by Orman and our study. First of all, Orman et al. analyzed oLT in a different region on a geographical as well as organizational level. While the US study involves UNOS data, our data are derived from a German center in the Eurotransplant region. The US system with the UNOS operated Organ Procurement and Transplant Network differs significantly from the European situation within the eight nations of the Eurotransplant region. The differences include a different allocation system, a different donor risk index within the donor population [24], as well as a different mean MELD score at the time of oLT. So far no studies exist investigating a potential influence of a weekend effect on outcomes in oLT in the Eurotransplant region. Our data reveal that there was no weekend effect on one-year allograft and patient survival, as well as no effect on short-term outcomes and surgical complications after oLT.

When comparing our results to other fields of surgery with respect to a possible weekend effect, one has to consider important differences between emergency surgery and solid organ transplantation. A unique feature of oLT is the non-existing differentiation of elective vs. emergency procedures. With the exception of living donation, there is no elective surgery in transplant surgery, which could be another explanation for an absent weekend effect in our cohort. It was suggested that patients admitted to hospitals on weekends or requiring surgery on weekends might suffer from more acute, life-threatening diseases, which in turn may confound differences in their outcomes [15]. In our cohort, we could exclude this factor since we found no significant differences in MELD score, HU-status or indication for oLT.

Another suggested explanation for a weekend effect is a critical reduction in personal resources as well as organizational and logistical differences. Thus, the results of our study must be interpreted in the context of staffing. Organs were recovered by regional professional procurement teams, which were not necessarily from our center. As for the transportation from donor hospitals to our center, logistical differences regarding traffic (road traffic, airplane availability etc.) were inevitable and could sometimes even be favorable during weekend time. However, our data on cold ischemia times suggests that there was no statistical significant difference in transportation time. The similar cold ischemia time between weekday and weekend groups might also exclude the possibility of a potential delay in recipient preparation due to personal or organizational shortcomings over weekends. At our hospital, the weekend nursing and anesthesia teams for transplant operations were recruited from weekend in-house teams and were thus not transplant-specific. With regard to the lead surgeon, our center has specific attending transplant surgeons, which are assisted by a surgery fellow or resident. In addition, in our center oLT is only performed by a very small group of surgeons (seven surgeons in total for the study period, with three to five surgeons per year, respectively) ensuring a high case number per surgeon and minimizing possible learning curves. However, we did not conduct a special analysis based on the preforming surgeon, nor were learning curves analyzed as a potential cofounder. For direct post-operative care, all patients were admitted to the intensive unit, led by a board-certified anesthesiologist. Thus, one can conclude that regarding the cohort of the present study, there was no reduction in staffing during weekends for teams participating in oLT.

When analyzing specific reasons for a potential weekend effect in transplant medicine, there is growing evidence that the acceptance or decline of an offered organ might constitute a relevant confounder since it is susceptible for the weekend effect, thus dependent on the timing of procurement [25, 26]. A recent study by Mohan et al. revealed a 16% increase in the odds of discard for kidney grafts when procured on a weekend compared to weekdays [26]. Cohen et al. could show that weekend kidney procurement was associated with significantly later acceptance or discard [25]. It is important to state, that the before mentioned studies were only referring to the UNOS kidney allocation system and that no data for liver transplantation in the Eurotransplant regions exists. However, these results could be evidence for a potential bias in such that weekend patients might receive organs of higher quality since the threshold for declining a liver graft might be lower on weekends if weekend transplant teams decline marginal organs or organ offers for high-risk candidates. While no available data was available on decline rates, our analysis shows that the frequencies for marginal liver grafts and high-risk recipients were comparable between weekend and weekday oLT and that no differences in DRI were detected suggesting a comparable donor quality during weekends and weekdays.

It is important to recognize some limitations of this study. First of all, we recognize that long-term five-year survival data would increase the merit of our study. In addition, we acknowledge that inherent to the study design we present single center data. Although we are aware of the growing body of evidence describing a weekend effect for various surgical specialties, we would assume that our single center results can be transferred to oLT in general, at least in Germany. Reasons for an absent weekend effect in the setting of oLT include standardized protocols, operationalized processes, experienced surgeons and highly trained interdisciplinary post-surgical care, all of which characterize transplant centers in general. A potential confounder could arise from weekday procedures on holidays when the organizational setting is more is more likely to resemble a weekend status. We found that within the weekend group no liver transplantation was conducted on a holiday while in the weekday group seven (2.8%) procedures were done on a holiday. However, even when these seven case were counted as weekend oLTs, the results remained consistent.

One has to consider additional steps which could be undertaken to further antagonize a possible weekend effect. One possibility would be to delay the organ procurement. However, while the data on this topic is conflicting, this might expose the procured organs to a greater damage due to brain death associated changes [27] and would further increase organizational and financial burdens for donor hospitals [28]. A more feasible approach would be to increase the awareness of a weekend effect among healthcare professionals, implement efficient, strategic and standardized protocols, provide sufficient staffing as well as set up and coordinate specialized multidisciplinary units. We hypothesize that all of the before mentioned is in part responsible for an absent weekend effect on oLT at our center. Thus, other disciplines might profit to adopt these strategies to weekend procedures to overcome a more evident risk for patients.

While we recognize some limitations, we believe that our results are of great merit for patients and transplant professionals. Especially for patients on the liver waiting list who cannot choose their date of surgery, these results should offer reassurance that they will receive excellent service throughout the week. Our results are encouraging and show that oLT is a safe, standardized and successful procedure at our center, independent from the day of the week. In conclusion, we provide the first retrospective single center data within the Eurotransplant region on oLT stratified for weekend and weekday procedures. While the weekend effect has been described for various time-sensitive acute conditions and emergencies, we did not observe a weekend effect on one-year patient survival graft survival or secondary outcomes such as complications requiring reoperation, re-transplantation or primary non-function. While we hypothesize that the absent weekend effect is due to standardized transplant procedures, qualified nursing staff and specialized multidisciplinary transplant teams, further studies are needed to understand this multifactorial phenomenon.
